# Morphometric Changes in Lateral Ventricles of Patients with Recent-Onset Type 2 Diabetes Mellitus

**DOI:** 10.1371/journal.pone.0060515

**Published:** 2013-04-04

**Authors:** Junghyun H. Lee, Sujung Yoon, Perry F. Renshaw, Tae-Suk Kim, Jiyoung J. Jung, Yera Choi, Binna N. Kim, Alan M. Jacobson, In Kyoon Lyoo

**Affiliations:** 1 Department of Pharmacy, Ewha University College of Pharmacy, Seoul, South Korea; 2 Department of Psychiatry, Catholic University of Korea School of Medicine, Seoul, South Korea; 3 The Brain Institute, University of Utah, Salt Lake City, Utah, United States of America; 4 Department of Psychiatry, University of Utah, Salt Lake City, Utah, United States of America; 5 Interdisciplinary Program in Neuroscience, Seoul National University College of Natural Sciences, Seoul, South Korea; 6 Research Institute, Winthrop University Hospital, Mineola, New York, United States of America; 7 Division of Life and Pharmaceutical Sciences, Ewha University Graduate School, Seoul, South Korea; 8 Ewha Brain Institute, Ewha University, Seoul, South Korea; Centre Hospitalier Universitaire Vaudois Lausanne-CHUV, UNIL, Switzerland

## Abstract

It is becoming increasingly evident that type 2 diabetes mellitus can have effects on global and regional brain morphology. Ventricular enlargement reflecting cerebral atrophy has been reported particularly in elderly type 2 diabetes patients. However, little is known about its timing through the disease course and morphological variability. Using the combined volumetric and advanced three-dimensional morphological approach, we identified differences in size and shape of the lateral ventricles between recent-onset type 2 diabetes patients and healthy individuals. High-resolution T1-weighted images were obtained from 23 type 2 diabetes patients whose illness duration was less than 1 year and 23 carefully matched healthy individuals. By volume measurement, we found enlarged lateral and third ventricles in type 2 diabetes patients, relative to healthy individuals (*F*_1,41_ = 7.96, *P* = 0.007; *F*_1,41_ = 11.16, *P* = 0.002, respectively). Morphological analysis revealed that the expansion of lateral ventricles in the diabetic brain was prominent in the bilateral frontal horns. The current findings suggest that atrophic changes particularly of the anterior frontal lobe can occur as early as the first year after the clinical diagnosis of type 2 diabetes mellitus.

## Introduction

Increasing evidence indicates that type 2 diabetes mellitus resulting from insulin insensitivity can give rise to a wide range of complications in the central nervous system (CNS), including cognitive decline and development of dementia [1]–[3]. As neuroanatomical correlates of these clinical manifestations, apparent structural changes in the brain have been documented by previous literature on neuroimaging of diabetes [4], [5]. A recent meta-analysis has suggested the association between type 2 diabetes mellitus and modest cerebral atrophy as the most convincing evidence [4]. White matter hyperintensity, despite the less robustness of findings, has also been observed in the diabetic brain [4].

Several demographic and metabolic risk factors have been supposed to be partly responsible for the type-2-diabetes-related brain abnormalities. Prolonged disease duration, comorbid cardiovascular diseases, and the extent of metabolic insults including hypo- and hyper-glycemia may contribute to the progress of cerebral atrophy in type 2 diabetes patients [6]–[9]. However, it is still unclear whether cerebral atrophic changes could begin early in the course of diabetes, even when these concomitant risk factors might not yet be prominent.

Abnormal ventricular enlargement has been considered as a surrogate marker of cerebral atrophy, partly due to the adaptive capacity of the fluid-filled ventricular system [10]. Furthermore, since the ventricular system is a relatively well-defined brain structure and could easily be segmented [11], it has widely been studied in association with several neuropsychiatric diseases. For example, ventricular enlargement has been reported as a major neuroimaging correlate of Alzheimer's dementia, a disease which is characterized by progressive cerebral atrophy during its disease course [10], [12]. However, only a few studies in elderly patients have heretofore been conducted to measure ventricular volume by employing segmentation followed by volumetric analysis of the lateral ventricles [7], [8], [13], [14]. Accordingly, further research is needed to demonstrate whether the diabetes-related brain changes would also be prominent in young and middle-aged patients, particularly when brain aging might not become evident.

Although the volumetric approach has been known as a natural and intuitive means to measure structural changes in the lateral ventricles and has therefore been widely used, it has limitations in providing specific anatomical information on regional changes [15]. More recently, three-dimensional shape analysis of the lateral ventricles has been developed for a more refined detection of subtle changes in shape composition, which could have been neglected in volumetric measurement [16]–[18]. As the expansion of the ventricular system may be a consequence of atrophic changes in nearby neuroanatomical structures [17], [18], diabetes-related alterations in the lateral ventricles could be assumed to begin at specific locations depending on cerebral regional susceptibility to metabolic insults. Therefore, three-dimensional shape analysis could enable to depict even subtle effects of diabetes on the structural variability of the lateral ventricles in a manner complementary to the volumetric approach. However, to the best of our knowledge, diabetes-related structural changes of the lateral ventricles have never been studied in terms of shape deformation.

In the study presented here, we examined volume differences in the ventricular system between young and middle-aged type 2 diabetes patients who had been diagnosed with diabetes less than 1 year ago and carefully matched healthy individuals. By using three-dimensional shape analysis, we further determined whether volumetric abnormalities of the lateral ventricles in the early stage of diabetes were localized. The information from the present study could provide important clues about the early influence of type 2 diabetes on neuroanatomical structures surrounding the lateral ventricles.

## Materials and Methods

### Ethics statement

The study protocol was approved by the institutional committee on human subjects of the Seoul National University Hospital and the St. Paul's Hospital of the Catholic University of Korea College of Medicine. All participants were given a complete explanation of the study details and then provided written informed consent to participate in the study.

### Subjects

A total of 23 patients with type 2 diabetes mellitus (mean 46.4 years, interquartile range = 38.1 to 53.1 years) and 23 healthy individuals (mean 45.3 years, interquartile range = 39.5 to 51.4 years) were enrolled in this study. Eligible patients were diagnosed with type 2 diabetes mellitus with duration of less than 1 year. The presence of serious diabetic complications including end-stage renal disease, painful and/or symptomatic neuropathy, and proliferative retinopathy was an exclusion criterion. Type 2 diabetes patients and healthy individuals who were enrolled in the study did not have any major medical, psychiatric, or neurological disorders. Although one patient had well-controlled hyperlipidemia, there were no subjects who had a current or past history of cardiovascular diseases including hypertension and coronary or carotid artery diseases. The presence of any contraindication to magnetic resonance (MR) imaging was another exclusion criterion for both type 2 diabetes patients and healthy individuals.

### Image Acquisition

Brain MR scans were acquired from subjects on the 1.5-Tesla whole-body imaging system (Signa HDx, GE Healthcare, Milwaukee, WI). Using a 3-dimensional spoiled gradient echo sequence, sagittal T1-weighted images were obtained (echo time TE = 5 ms, repetition time TR = 24 ms, matrix = 256×192, field of view FOV = 24 cm, flip angle FA = 45°, number of excitation NEX = 2, slice thickness = 1.2 mm, no skip). Axial T2-weighted images (TE = 126 ms, TR = 2,817 ms, matrix = 256×192, FOV = 22 cm, FA = 90°, NEX = 1, slice thickness = 5 mm, no skip) and fluid-attenuated inversion recovery (FLAIR) axial images (TE = 88 ms, TR = 8,802 ms, TI = 2,200 ms, matrix = 256×192, FOV = 22 cm, FA = 90°, NEX = 1, slice thickness = 5 mm, no skip) were also obtained.

All images were examined for gross structural abnormalities by a board-certified neuroradiologist who did not know subjects' diagnosis or clinical information.

### Image Analysis: Segmentation and Shape Analysis of the Ventricles

For the segmentation of the ventricular system, high-resolution T1-weighted images were processed using a rater-independent, automated atlas-based tissue segmentation method implemented in the FreeSurfer software (http://surfer.nmr.mgh.harvard.edu) [19]. In brief, after the processing of motion correction, intensity inhomogeneity correction, and the removal of non-brain tissue, each ventricular component was segmented by assigning each voxel of the preprocessed volume to the corresponding ventricle labels. Information about voxel intensity relative to probabilistic training atlas was utilized for these assignments and subsequent comparisons to neighboring voxel label obtained from atlas were performed [19]. Final segmented images were visually inspected by an expert who judged the appropriateness of images in further shape analysis. Intracranial volume (ICV) and volumes of each ventricular component were measured. As exploratory analyses, cortical regions were segmented based on the Desikan-Killiany atlas [20] and volumes of each cortical region were measured using the FreesSurfer software.

The spherical harmonic-based shape description was used for shape modeling of the lateral ventricles. The semi-automated processing for shape description was performed with the use of the University of North Carolina shape analysis toolkit [15], [16]. In brief, binary segmented images of the lateral ventricle were converted to surface meshes and spherical parameterization was computed [21]. Spherical harmonic correspondence between vertices of surface meshes was established by using parameter-based rotation based on the first order ellipsoid from the spherical harmonic coefficients [22]. This step could eliminate the effects of rotation and translation. The spherical harmonic description was then uniformly sampled into a 2252 triangulated surface points. The surfaces of lateral ventricles were spatially aligned to averaged template surface using a rigid-body transformation [23]. In this analysis, the frontal horn and main body of the lateral ventricles were included in the analysis, because connections with the temporal horn could not occasionally be resolved even in high-resolution MR images [16].

### Statistical Analysis

Demographic and clinical characteristics were compared between groups using independent t-tests or chi-square tests for continuous and categorical variables, respectively.

For the comparison of volume measurements of each ventricular component of the third, fourth, and the total lateral ventricles, analysis of covariance (ANCOVA) was used to examine the differences in ventricular volumes between type 2 diabetes patients and healthy subjects adjusting for age, sex, and ICV. Standardized volume indices (z scores) for each ventricular component were computed to estimate the effect size of group differences in volume. *Z* scores were calculated by using the mean and standard deviation (SD) of each ventricular volume of healthy individuals and adjusted for age, sex, and ICV. Positive z scores represent the number of SD units of volume enlargement relative to mean ventricular volume of healthy individuals.

For complementary information on cortical volume measurements, ANCOVA was used to examine the group differences in volumes of each cortical region adjusting for age, sex, and ICV. In addition, partial correlation analyses including age, sex, and ICV as covariates were performed in order to determine cortical regions in which volume reductions were associated with lateral ventricular enlargement in each group.

Partial correlation analyses were performed to examine the relationships of ventricular volumes with either disease duration or hemoglobin A1C (HbA_1c_) after covarying for age, sex, and ICV.

For the statistical analysis of surface coordinates at 2252 corresponding points in each left and right lateral ventricles, multivariate analysis of covariance with Wilk's lambda was used to assess the diagnostic effects on surface coordinates adjusting for age, sex, and ICV. In order to correct for multiple comparisons in imaging data, surface-based cluster size exclusion was used with an initial surface-vertexwise threshold of *P*<0.001. Any difference in surface multivariate metric within a cluster containing less than 5 vertices was excluded. This threshold was ensured to be in corrected *P* value of <0.05 across the surface of each ventricle by using the method of AFNI's AlphaSim [24], which was adapted for use with surface-based statistics [25].

All statistical analyses were performed using Stata SE, v12.0 (Stata Corp, College Station, TX).

## Results

Type 2 diabetes patients and healthy individuals were well matched in terms of age and sex. Demographic and clinical characteristics of study subjects are presented in Table 1.

**Table 1:** Demographic and clinical characteristics of patients with type 2 diabetes mellitus and healthy individuals.

Characteristics	Type 2 diabetes patients (n = 23)	Healthy individuals (n = 23)
***Demographic characteristics***		
Age, mean (SD), y	46.4 (8.3)	45.3 (7.3)
Female, n (%)	10 (43.5)	10 (43.5)
***Diabetes-related characteristics***		
Time since diagnosis of diabetes, mean (SD), months	4.37 (3.25)	NA
Age at diagnosis of diabetes, mean (SD), y	46.0 (8.3)	NA
Blood glucose, mean (SD), mg/dL		
Fasting	143.2 (59.6)	97.1 (5.0)
Postprandial^a^	178.1 (104.1)	122.5 (5.5)
Glycated hemoglobin, mean (SD), %	7.49 (2.12)	5.45 (0.25)
Laboratory findings		
Urea nitrogen, mean (SD), mg/dL	14.5 (3.6)	14.0 (3.3)
Creatinine, mean (SD), mg/dL	0.87 (0.14)	0.90 (0.16)
Sodium, mean (SD), mEq/L	141.6 (2.3)	141.6 (2.1)
Potassium, mean (SD), mEq/L	4.22 (0.25)	4.17 (0.42)
Hemoglobin, mean (SD), g/dL	14.4 (1.5)	14.4 (1.3)
Hematocrit, mean (SD), %	41.0 (4.3)	41.4 (4.2)

a Data from one patient with type 2 diabetes mellitus are not available.

Abbreviations: SD, standard deviation; NA, not available.

All patients were diagnosed as having type 2 diabetes mellitus at the University-affiliated hospital according to the American Diabetes Association criteria [26], and the mean time since the clinical diagnosis of type 2 diabetes mellitus was 4.36 months (SD = 3.25 months, range = 0.5 to 11.9 months), which indicated that the participating type 2 diabetes patients were in a relatively early stage of illness. Two patients (8.7%) had only dietary management, while 21 subjects (91.3%) received oral hypoglycemic agents. Patients did not have any history of hypoglycemic episodes. Glycemic control levels of all healthy individuals were within normal range (fasting blood glucose levels, mean 97.1 mg/dl, range = 87.0 to 106.0 mg/dl; HbA_1c_ levels, mean 5.45%, range = 5.10 to 5.80%). Blood glucose (fasting, *t* = −3.70, *P*<0.001; postprandial, *t* = −2.56, *P* = 0.01) and HbA_1c_ levels (*t* = −4.57, *P*<0.001) were higher in type 2 diabetes patients than in healthy individuals. Other laboratory findings were not different between the groups (Table 1).

**Figure 1:**
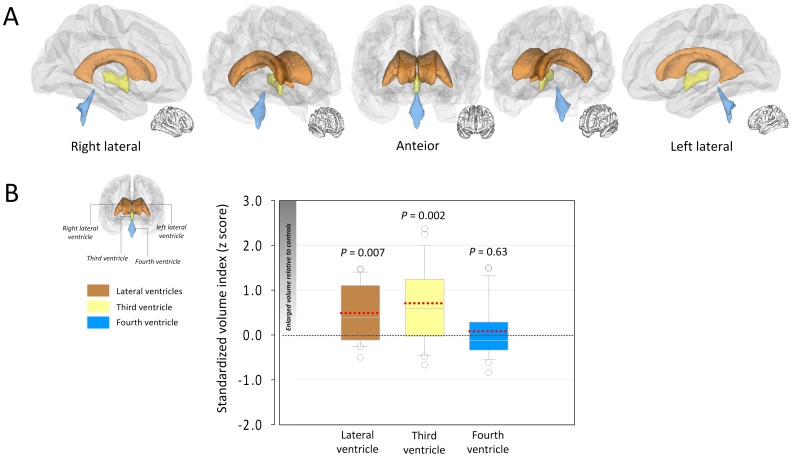
Anatomical location of the ventricles (A) and alterations of ventricular volumes (B) in recent-onset type 2 diabetes. Three-dimensional illustration of anatomical location for cerebral ventricles is presented in the Panel A. The box-and-whisker plot in the Panel B presents the standardized volume index (z scores) for each ventricular component of type 2 diabetes patients. *Z* scores were calculated by using the mean and standard deviation of each ventricular volume of healthy individuals and adjusting for age, sex, and intracranial volume. Positive z scores indicate enlarged ventricular volumes. Dotted red lines indicate the means of the respective z scores. Analysis of covariance showed significant volume enlargements of the lateral and the third ventricles in type 2 diabetes patients relative to healthy individuals (lateral ventricles, *F*_1,41_ = 7.96, *P* = 0.007; third ventricle, *F*_1,41_ = 11.16, *P* = 0.002).

Table 2 shows adjusted volumes of each ventricular component. There was no overall difference in ICV between groups (*F*_1,42_ = 0.05, *P* = 0.82). Patients with type 2 diabetes mellitus showed volume enlargements in the lateral and third ventricles relative to healthy individuals after covarying for age, sex, and ICV (*F*_1,41_ = 7.96, *P* = 0.007 and *F*_1,41_ = 11.16, *P* = 0.002, respectively). Standardized volume indices by z scorization for the lateral and the third ventricles were 0.97 and 1.41, respectively (Figure 1). Volumes of the fourth ventricle were not different between groups (*F*_1,41_ = 0.23, *P* = 0.63).

**Table 2:** Volumetric measurements of patients with type 2 diabetes mellitus and healthy individuals.

Ventricular system	Type 2 diabetes patients (n = 23)	Healthy individuals (n = 23)
	mean (SD)	mean (SD)
Intracranial volume, cm^3^	1437.3 (133.3)	1444.6 (108.1)
Right lateral ventricle, a mm^3^	6338.5 (1376.7)	5608.3 (1310.8)
Left lateral ventricle, a mm^3^	7624.9 (1835.5)	6047.2 (1214.5)
Total lateral ventricle, a mm^3^	13963.3 (2973.3)	11655.4 (2368.1)
Third ventricle, a mm^3^	1123.7 (255.2)	910.5 (151.3)
Fourth ventricle, a mm^3^	1852.7 (443.3)	1793.6 (356.9)

a Volumes were adjusted for age, sex, and intracranial volume.

Abbreviations: SD, standard deviation.

Since recent experimental studies have suggested the effects of acute dehydration state on the ventricular volumes [27]–[31], we performed repeated analyses including blood urea nitrogen, creatinine, sodium, potassium, fasting glucose, or hematocrit levels, which could potentially reflect subjects' hydration state [32]–[36], as covariates in order to ensure the robustness of our findings. Similar results were produced showing that the ventricular enlargement in type 2 diabetes patients were not likely to be the only attribution to the dehydration state (Table S1).

Results from exploratory analyses on cortical volume measurement are presented in Figures S1 and S2.

Partial correlation analyses demonstrated a trend of positive association of lateral ventricular enlargement and disease duration which did not, however, reach the statistical significance (*r* = 0.40, *P* = 0.079). There is no relationship between lateral ventricular volume and HbA_1c_ levels (*r* = −0.27, *P* = 0.25). The volume of the third ventricle was correlated neither with disease duration (*r* = 0.26, *P* = 0.26) nor with HbA_1c_ levels (*r* = −0.36, *P* = 0.12).

**Figure 2:**
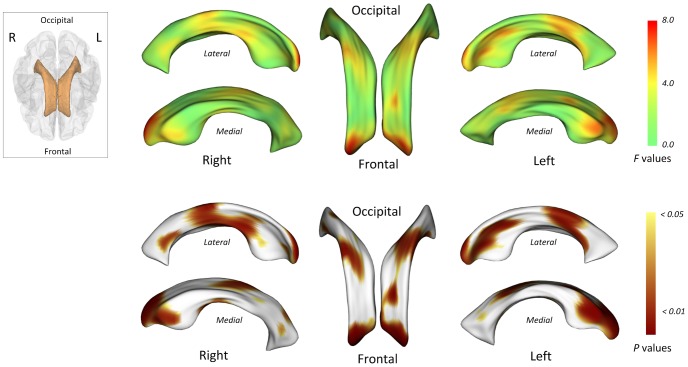
Locations of shape differences in the lateral ventricles, uncorrected for multiple comparisons. The F statistic (upper column) and probability (lower column) maps show the results of multivariate analysis of covariance (MANCOVA) for estimating the group differences in surface coordinates after adjusting for age, sex, and intracranial volumes.

**Figure 3:**
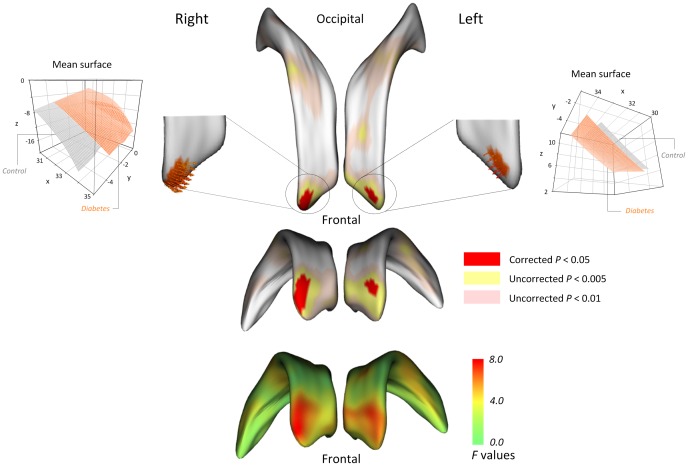
Locations of shape differences in the lateral ventricles, corrected for multiple comparisons. To correct for multiple comparisons, surface-based cluster-size exclusion with the initial threshold of *P*<0.001 was used. Any difference in surface metric within a cluster containing less than 5 vertices was excluded. After the corrections for multiple comparisons, the expansion of lateral ventricles in type 2 diabetes patients was prominent in the bilateral frontal horns of the lateral ventricles (regions in red color).

Statistical maps for comparisons of surface coordinates of the bilateral lateral ventricles are presented in Figure 2. All maps were adjusted for age, sex, and ICV. There appear to be bilateral ventricular expansion in type 2 diabetes patients, particularly of the frontal horns and the mid-portion relative to healthy individuals (red color in *F* statistic map and brown color in *P* map of Figure 2). After the correction for multiple comparisons (corrected *P*<0.05), type 2 diabetes patients showed a significant localized expansion of the frontal horns of the bilateral lateral ventricles to the surrounding brain regions including the medial frontal lobe (Figure 3).

## Discussion

This is the first study to examine the effects of type 2 diabetes mellitus on lateral ventricular morphometric abnormalities using three-dimensional shape analysis complementary to conventional volumetric approach. We found that type 2 diabetes patients showed a significant volume enlargement of the lateral ventricles relative to healthy individuals, despite a relatively short illness duration (mean time since the clinical diagnosis of type 2 diabetes mellitus, 4.37 months). Shape analysis revealed that these abnormalities related to diabetes were highly localized in the bilateral frontal horns of the lateral ventricles.

Lateral ventricular enlargement remains one of the most robust brain abnormalities in Alzheimer's dementia [10], [12]. Not only volume expansion but also the rate of changes in ventricular volume has been studied as a potential surrogate marker to predict the development and progression of this type of dementia [10]. Since the empirical evidence on the possible link between type 2 diabetes mellitus and Alzheimer's dementia has been suggested [1], [2], research efforts have focused to find relevant neuroimaging correlates of this relationship particularly in elderly type 2 diabetes patients [7]–[9], [14], [37], [38]. Most recently, a series of longitudinal observations have indicated that type 2 diabetes patients revealed progressive lateral ventricular enlargement at a rate exceeding that of normal aging [7], [39], [40] and this abnormality might be correlated with the extent of patients' cognitive decline [14], [40]. These findings strongly support that type 2 diabetes mellitus might accelerate age-related brain changes such as ventricular enlargement that could possibly confer increased risks of dementia in diabetes patients. The current findings extend the body of evidence supporting that the acceleration of brain aging by diabetes could begin at an early stage of disease even in middle-aged patients. Likewise, a recent neuroimaging study suggests that not only diabetes but also the prediabetic state could influence subclinical changes in brain volumes [41]. Furthermore, our findings are consistent with the previous findings of several research groups, which suggest subclinical cognitive impairment in recent-onset diabetes as well as in the prediabetic state [41]–[43].

Several cellular mechanisms have been inferred to underlie the structural changes in the diabetic brains. Hyperglycemia and abnormal insulin metabolism altogether with vascular lesions might contribute to the cerebral neuropathology related to type 2 diabetes mellitus [1]. It is notable that several of these mechanisms related to prolonged hyperglycemia, such as oxidative stress and advanced glycation end-products accumulation, have also been implicated in the brain aging process [1], [2]. Furthermore, recent studies suggested that acute dehydration state, which could occur concomitantly with type 2 diabetes mellitus, might be related to the brain atrophic changes and ventricular enlargement [27]–[31]. Despite similar laboratory profiles of hydration state between the groups and consistent results even after covarying potentially confounding factors related to subjects' hydration state, a subclinical dehydration condition related to type 2 diabetes mellitus could contribute to ventricular enlargement in an early stage of disease.

Even though it is unclear which mechanisms might play a major role particularly in early structural changes of the diabetic brain, the current findings might provide important insights into the timing of the onset of neuropathological abnormalities related to type 2 diabetes mellitus.

Although the progressive volume enlargement of lateral ventricles over the disease course has been reported [7], [39], [40], we did not find significant associations of ventricular volumes either with disease duration or with glycemic control levels. In part, a relatively small sample size might have imposed limitation on demonstrating the relationship between the overall disease burden and ventricular enlargement. Furthermore, among our sample, very recent-onset patients (illness duration <3 months, n = 11, mean HbA_1C_ = 8.77%) whose glycemic control levels could not be stabilized yet, had higher HbA_1C_ levels than early-stage patients (6.31% of remaining 12 patients). These sample characteristics might have hampered the identification of potential dose-responsive effects of diabetes on ventricular enlargement. Future research efforts to find potential vascular and metabolic factors that could be associated with accelerated global and regional atrophic changes of early type 2 diabetes would be necessary.

The lateral ventricles are the largest in the ventricular system extending from the frontal to temporo-occipital lobes [44]. Accordingly, region-specific patterns of the affected lateral ventricles might be subordinate to the structural changes in nearby subcortical structures and cortices. For example, the expansion of the anterior portion of the lateral ventricles is likely to reflect atrophic changes of the frontal lobe and the basal ganglia, while the involvement in the posterior portion could be associated with changes in the parietal or occipital lobes. Our three-dimensional shape analysis revealed a significant expansion of the frontal horns of the lateral ventricle in type 2 diabetes patients, indirectly suggesting a concomitant volume decrease in the medial frontal region. Although previous neuroimaging on type 2 diabetes mellitus has mainly focused on whole brain atrophic changes [4], [5], a limited number of recent studies have reported the disproportionately region-specific involvements of the diabetic brain. The medial temporal regions including the hippocampus, in which insulin receptors are abundantly located, have first received attention in studies of elderly type 2 diabetes patients with a mean age of over 60 years, as these regional involvements might also be pronounced in Alzheimer's dementia [45], [46]. More recently, abnormalities in the medial frontal regions, which have reciprocal connections with the subcortical structures including the basal ganglia and the hippocampus, have been reported in middle-aged diabetes patients [6], [47], [48]. Not only medial frontal gray matter volume reductions but also altered white matter connectivity in these regions was observed in type 2 diabetes patients [6], [47], [48]. Together with these previous observations, the present findings on selective expansion of the frontal horns of the lateral ventricles provide a new understanding that the abnormalities in the frontal lobe might occur early in the course of type 2 diabetes mellitus. As suggested by the critical role of the frontal lobe in the regulation of mood and cognition [47], [49], the earlier involvement of the frontal lobe in type 2 diabetes mellitus might be important in accounting for frequently comorbid depression and cognitive decline in this metabolic disease.

It is notable that third ventricle enlargement was also found in type 2 diabetes patients. Anatomically, the third ventricle communicates with the lateral ventricle through the interventricular foramen of Monro and is bound by the nuclear (the thalamus and the hypothalamus) and glandular (the pituitary and pineal glands) structures [44], [50]. With an understanding of the anatomical location of the third ventricle, our findings could be understood as to propose that abnormalities in these adjacent structures may potentially occur in association with type 2 diabetes mellitus. In any case, the present finding requires careful replications in type 2 diabetes patients, with assessments of the targeted brain structures.

There are several limitations of the present study. The current three-dimensional shape analysis could have unique methodological advantages in identifying the focal region-specific disease-associated alterations in the lateral ventricles [16]–[18]. However, since the temporal horns of the lateral ventricles were excluded from the analysis as in other numerous studies using the morphometric approach to the lateral ventricles [16], [18], [51], the current results should be interpreted in relation to the focused regions that could be included in our analysis. For instance, temporal regional deficit, which have been reported in the diabetic brain [45], [46], could not be determined in this study. In a related limitation, although our exploratory findings indicated that lateral ventricular enlargement in type 2 diabetes patients might reflect volume loss in the lateral orbitofrontal, inferior frontal, and temporal regions (Figure S2), we only found trend-level region-specific differences in cortical volumes between type 2 diabetes patients and healthy subjects (Figure S1), in part attributable to a relatively small sample size as well as small to medium effect sizes of type 2 diabetes-related cortical volume deficits. Future studies using more sensitive and direct measurements of cortical and subcortical regions will be necessary in a larger sample to detect early structural changes related to type 2 diabetes mellitus, which we could not find.

Because diabetes usually develops in a slow, insidious manner and often goes undiagnosed at least during the first years since the actual onset [7], obtaining the accurate estimates of illness duration in diabetes patients has certain difficulties. Although our subjects had no specific diabetes-related complications and appeared to be in the early stage of illness, future longitudinal observations to portray the trajectory of lateral ventricular enlargement will be warranted in a larger sample including subjects at the stage before the clinical diagnosis of type 2 diabetes mellitus, to confirm the early brain changes related to metabolic insults.

In conclusion, our study using the advanced three-dimensional morphometric analysis along with conventional volumetric approach not only strengthens earlier findings indicating accelerated brain aging related to type 2 diabetes but first reports that associated lateral ventricular enlargement could begin in the early stage of type 2 diabetes mellitus. Furthermore, given the previous compelling evidence on the strong interaction effect of aging and type 2 diabetes mellitus on the brain and cognition [52], the current findings raise intriguing issues, as they suggest brain changes related to type 2 diabetes mellitus even when the aging process of the brain might not become evident. The current results have clinical implications to highlight the importance of controlling metabolic abnormalities in preventing future dementia as early as possible, after the diagnosis of type 2 diabetes mellitus or even in the prediabetic state.

## Supporting Information

Figure S1 **Comparisons of volumes in the parcellated cortical regions based on the Desikan-Killiany atlas between type 2 diabetes patients and healthy individuals.** Analyses of covariance were performed to examine the group differences in cortical volumes after adjusting for age, sex, and intracranial volumes. There were no regions of significant volume differences between type 2 diabetes patients and healthy individuals. For the descriptive purpose, regions of volume reductions in type 2 diabetes patients relative to healthy individuals at a trend-level of statistical significance are presented with asterisk (* P<0.10). We found that type 2 diabetes patients showed superior temporal (F1,41 = 3.43, P = 0.071) and inferior parietal (F1,41 = 3.07, P = 0.087) cortical volume reductions relative to healthy individuals.
(TIF)

Figure S2 **Cortical regions showing volume reductions associated with lateral ventricular enlargement.** Partial correlation analyses including age, sex, and intracranial volume as covariates were performed to examine the relationships between regional cortical volume reduction and lateral ventricular enlargement in each type 2 diabetes mellitus and control group. Note that cortical volume in the inferior frontal, orbitofrontal, and the temporal regions were negatively associated with the lateral ventricular volume in type 2 diabetes patients (pars opercularis, r = −0.59, P = 0.006; pars orbitalis, r = −0.62, P = 0.003; lateral orbitofrontal, r = −0.55, P = 0.011; superior temporal, r = −0.50, P = 0.025; middle temporal, r = −0.46, P = 0.039; fusiform, r = −0.52, P = 0.019) but not in healthy subjects (pars opercularis, r = −0.09, P = 0.71; pars orbitalis, r = −0.11, P = 0.65; lateral orbitofrontal, r = −0.37, P = 0.10; superior temporal, r = −0.12, P = 0.61; middle temporal, r = 0.15, P = 0.54; fusiform, r = 0.23, P = 0.33). There were no regions of significant volume increase in association with lateral ventricular enlargement both in the type 2 diabetes and control groups. Abbreviation: T2DM, type 2 diabetes mellitus.
(TIF)

Table S1 **Results from the repeated analyses for group differences in ventricular volumes including potential confounding factors as covariates.**
(DOC)
