# Supplementary figures and images for: Morphometric Changes in Lateral Ventricles of Patients with Recent-Onset Type 2 Diabetes Mellitus
Source: PLoS One. 2013 Apr 4;8(4):e60515. doi: 10.1371/journal.pone.0060515 (PMC3617143; doi:10.1371/journal.pone.0060515)

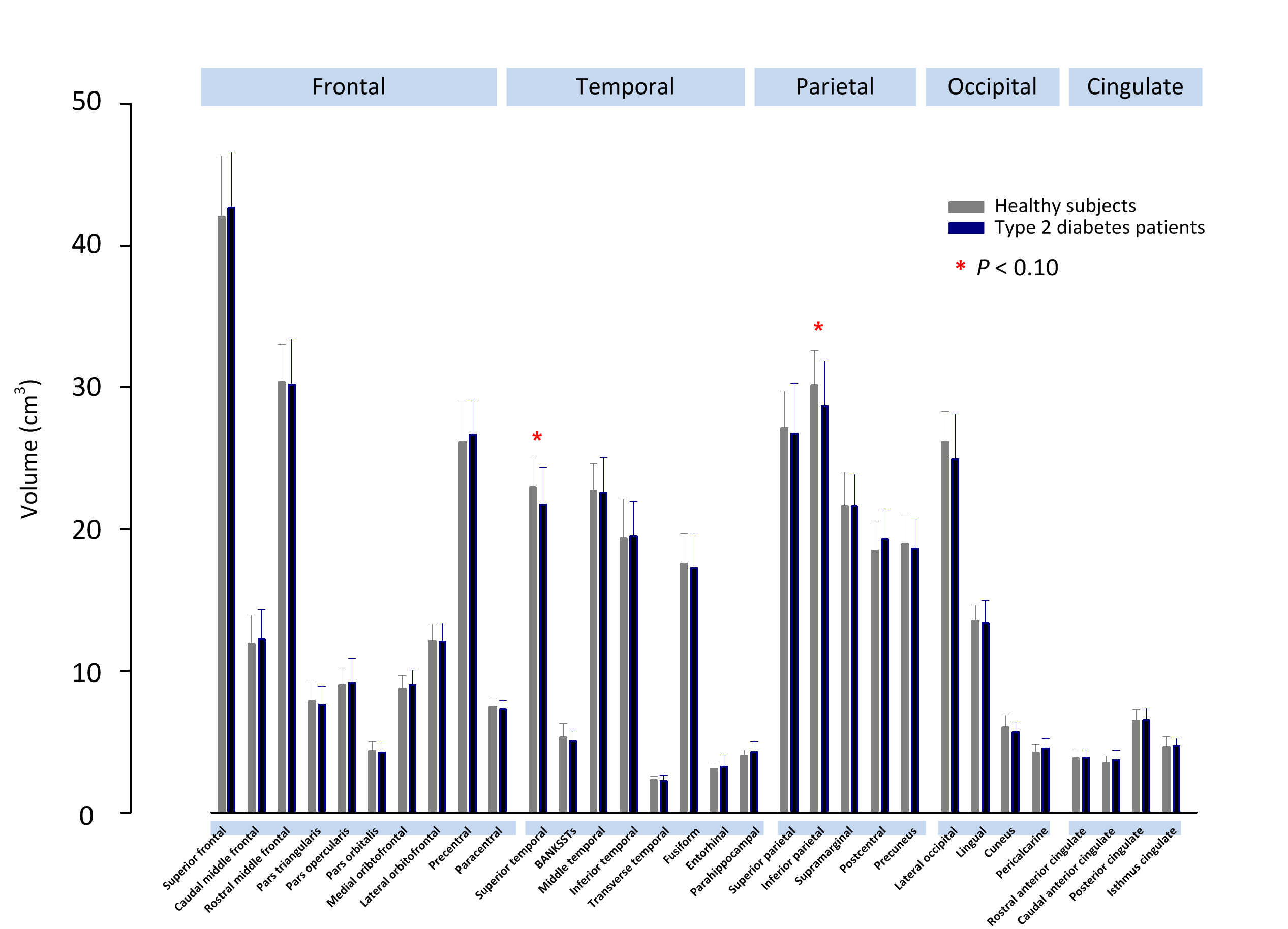

Supplement: Figure S1 — Comparisons of volumes in the parcellated cortical regions based on the Desikan-Killiany atlas between type 2 diabetes patients and healthy individuals. Analyses of covariance were performed to examine the group differences in cortical volumes after adjusting for age, sex, and intracranial volumes. There were no regions of significant volume differences between type 2 diabetes patients and healthy individuals. For the descriptive purpose, regions of volume reductions in type 2 diabetes patients relative to healthy individuals at a trend-level of statistical significance are presented with asterisk (* P<0.10). We found that type 2 diabetes patients showed superior temporal (F1,41 = 3.43, P = 0.071) and inferior parietal (F1,41 = 3.07, P = 0.087) cortical volume reductions relative to healthy individuals. (TIF) [file pone.0060515.s001.tif]

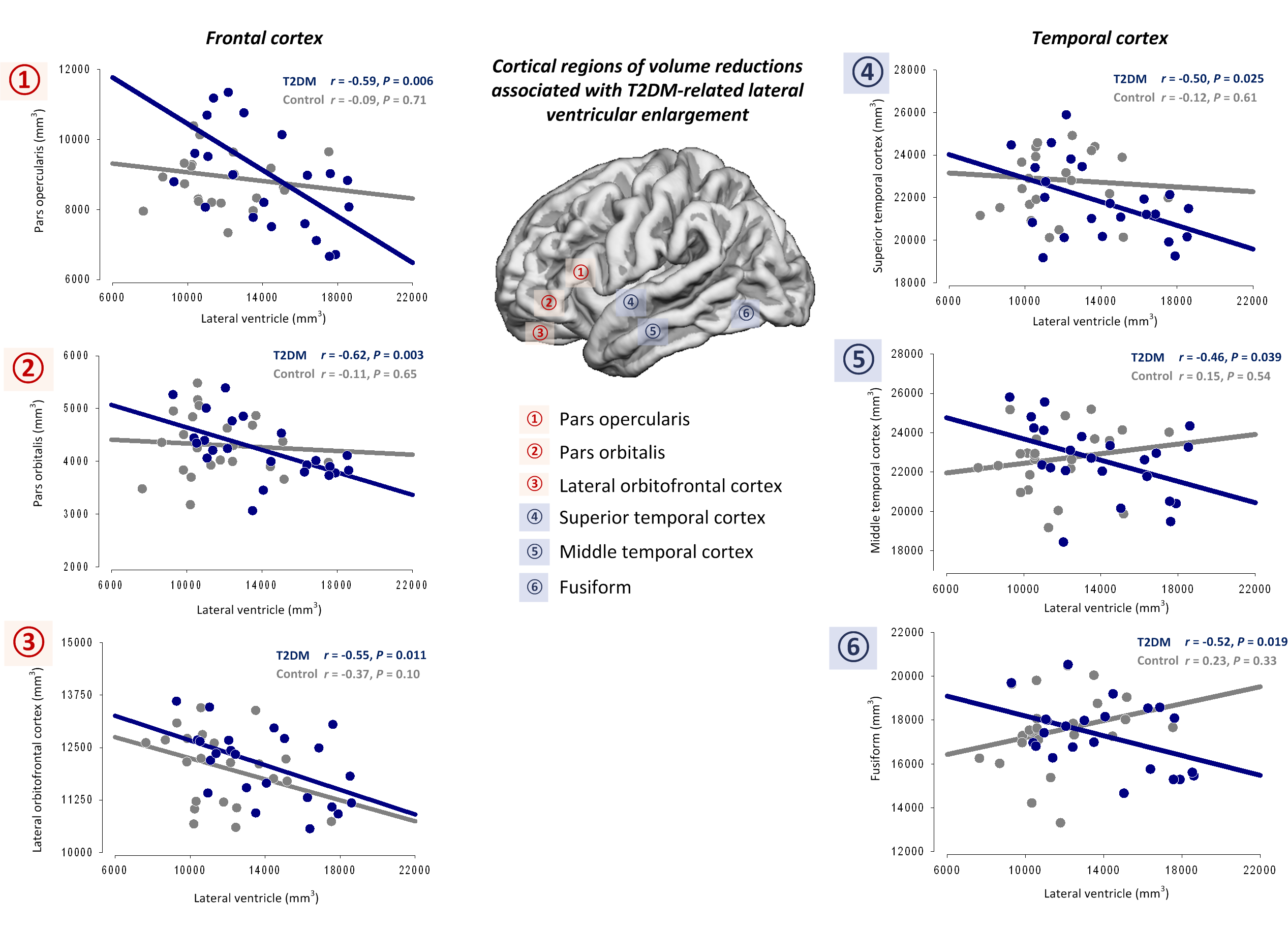

Supplement: Figure S2 — Cortical regions showing volume reductions associated with lateral ventricular enlargement. Partial correlation analyses including age, sex, and intracranial volume as covariates were performed to examine the relationships between regional cortical volume reduction and lateral ventricular enlargement in each type 2 diabetes mellitus and control group. Note that cortical volume in the inferior frontal, orbitofrontal, and the temporal regions were negatively associated with the lateral ventricular volume in type 2 diabetes patients (pars opercularis, r = −0.59, P = 0.006; pars orbitalis, r = −0.62, P = 0.003; lateral orbitofrontal, r = −0.55, P = 0.011; superior temporal, r = −0.50, P = 0.025; middle temporal, r = −0.46, P = 0.039; fusiform, r = −0.52, P = 0.019) but not in healthy subjects (pars opercularis, r = −0.09, P = 0.71; pars orbitalis, r = −0.11, P = 0.65; lateral orbitofrontal, r = −0.37, P = 0.10; superior temporal, r = −0.12, P = 0.61; middle temporal, r = 0.15, P = 0.54; fusiform, r = 0.23, P = 0.33). There were no regions of significant volume increase in association with lateral ventricular enlargement both in the type 2 diabetes and control groups. Abbreviation: T2DM, type 2 diabetes mellitus. (TIF) [file pone.0060515.s002.tif]
