# Supplementary material for: Morphometric Changes in Lateral Ventricles of Patients with Recent-Onset Type 2 Diabetes Mellitus
Source: PLoS One. 2013 Apr 4;8(4):e60515. doi: 10.1371/journal.pone.0060515 (PMC3617143; doi:10.1371/journal.pone.0060515)
Supplement: Table S1 — Results from the repeated analyses for group differences in ventricular volumes including potential confounding factors as covariates. (DOC) [file pone.0060515.s003.doc]

| **Table S1.** Results from the repeated analyses for group differences in ventricular volumes including potential confounding factors as covariates. | | | |
| --- | --- | --- | --- |
|  | **Volume differences between type 2 diabetes mellitus and healthy individuals** | | |
|  | **Total lateral ventricle** | **Third ventricle** | **Fourth ventricle** |
| Original findings ^a^ | *F*_1,41_ = 7.96, *P* = 0.007 | *F*_1,41_ = 11.2, *P* = 0.002 | *F*_1,41_ = 0.23, *P* = 0.63 |
| Repeated analysis including an additional covariate | | | |
| urea nitrogen level | *F*_1,40_ = 7.77, *P* = 0.008 | *F*_1,40_ = 10.8, *P* = 0.002 | *F*_1,40_ = 0.26, *P* = 0.61 |
| creatinine level | *F*_1,40_ = 10.9, *P* = 0.002 | *F*_1,40_ = 15.6, *P* < 0.001 | *F*_1,40_ = 0.19, *P* = 0.66 |
| sodium level | *F*_1,40_ = 7.76, *P* = 0.008 | *F*_1,40_ = 11.0, *P* = 0.002 | *F*_1,40_ = 0.24, *P* = 0.62 |
| potassium level | *F*_1,40_ = 7.40, *P* = 0.010 | *F*_1,40_ = 11.3, *P* = 0.002 | *F*_1,40_ = 0.13, *P* = 0.72 |
| fasting glucose level | *F*_1,40_ = 6.55, *P* = 0.014 | *F*_1,40_ = 11.2, *P* = 0.002 | *F*_1,40_ = 0.89, *P* = 0.35 |
| hematocrit level | *F*_1,40_ = 7.41, *P* = 0.010 | *F*_1,40_ = 10.9, *P* = 0.002 | *F*_1,40_ = 0.46, *P* = 0.50 |
| ^a^ Analyses of covariance were used to examine the differences in ventricular volumes between type 2 diabetes patients and healthy subjects adjusting for age, sex, and intracranial volume. | | | |
